# Humoral Response Following Triple Dose of mRNA Vaccines Against SARS-CoV-2 in Hemodialysis Patients: Results After 1 Year of Follow-Up

**DOI:** 10.3389/fmed.2022.927546

**Published:** 2022-07-12

**Authors:** Eduardo Gallego-Valcarce, Amir Shabaka, Mariana Leon-Poo, Enrique Gruss, Juan Manuel Acedo-Sanz, Alfredo Cordón, Clara Cases-Corona, Gema Fernandez-Juarez

**Affiliations:** ^1^Department of Nephrology, Hospital Universitario Fundación Alcorcón, Alcorcón, Spain; ^2^Department of Clinical Analysis, Hospital Universitario Fundación Alcorcón, Alcorcón, Spain; ^3^Centro de Diálisis Los Llanos, Fundación Renal Íñigo Álvarez de Toledo, Móstoles, Spain

**Keywords:** COVID-19, chronic kidney disease, immunology, vaccine, prevention

## Abstract

**Introduction:**

COVID-19 is associated with an increased mortality in hemodialysis patients. Therefore, achieving a long-lasting effective immune response to SARS-CoV-2 vaccines is essential. This study describes the humoral immune response in hemodialysis patients following three doses of mRNA vaccines against SARS-CoV-2, and explores the factors associated with a sustained immune response.

**Materials and Methods:**

We analyzed the monthly serological evolution of SARS-CoV-2 anti-S(RBD) antibodies for 1 year in 178 chronic hemodialysis patients who received three doses of SARS-CoV-2 mRNA vaccines. The primary outcome was sustained effective humoral response defined as anti-S(RBD) levels > 1,000 AU/ml after 4 months from the third dose. Multivariate logistic regression analyses were used to identify features associated with a sustained humoral immune response.

**Results:**

After the initial two SARS-CoV-2 mRNA vaccine doses, 77.8% of patients showed an immediate effective humoral response, decreasing to 52.5% after 4 months. Antibody levels were significantly higher in COVID-exposed patients and HBV vaccine responders. After the third dose, 97% of patients showed an effective humoral response, and remained in 91.7% after 4 months. The mean monthly rate of antibody titer decline decreased from 33 ± 14.5 to 25 ± 16.7%. Multivariate regression analysis showed that previous exposure to COVID-19 and response to HBV vaccines were associated with an effective sustained humoral immune response.

**Conclusion:**

Immunization with SARS-CoV-2 mRNA vaccines elicits an effective immediate humoral immune response in hemodialysis patients, with a progressive waning in antibody levels. A third booster dose enhances the immune response with significantly higher antibody levels and more sustained humoral immune response. COVID-naïve patients and patients without previous response to HBV vaccines are likely to benefit from receiving more booster doses to maintain an effective immune response.

## Introduction

Patients on hemodialysis (HD) have an increased incidence of infection with severe acute respiratory syndrome coronavirus 2 (SARS-CoV-2) compared to the general population, and when infected their mortality risk is higher (1–3). For these reasons, HD patients have been included as one of the priority groups in vaccination programs (4, 5). This practice has been carried out despite the fact that chronic kidney disease patients have been excluded from the main studies on vaccination (6–8) and that their immune response to vaccination against other viruses is less effective than in the general population (9).

There are no specific recommendations concerning the most adequate vaccine regimen for HD patients. Around 90% of HD patients without previous infection with SARS-CoV-2 develop an immediate humoral and cellular immune response after the administration of two doses of mRNA vaccines (10, 11). Despite these results, there is a reported mortality of 11% in HD patients who get infected with COVID-19 after receiving the two mRNA vaccine doses (12). This elevated mortality can be attributed to different factors: a lower and less effective immunological response compared to the general population (13, 14) as well as a progressive decrease in antibody levels (15). For these reasons, the administration of a third SARS-CoV-2 mRNA vaccine dose has been recommended (12, 16, 17).

There are no longitudinal studies concerning SARS-CoV-2 vaccine-induced immune responses for more than 4 weeks after receiving the third mRNA vaccine dose in patients on HD, which are needed to optimize clinical care and plan preventive strategies in this population.

In this study, we describe the serological monthly changes against SARS-CoV-2 in HD patients after 1 year of follow-up from an initial immunization with two doses of mRNA vaccines. A third booster dose was administered after 5 months from the second dose, with continuous monthly serological assessment for 4 months following the third vaccine dose. We aimed to describe the factors associated with a sustained humoral response and identify the group of patients who are more likely to benefit from receiving a third booster dose.

## Materials and Methods

### Study Design and Population

We designed an observational, longitudinal study to evaluate the immune response induced by the different mRNA vaccines against SARS-CoV-2 in patients attending two HD units: an in-center hospital dialysis unit and its affiliated satellite dialysis unit. The study comprised all HD patients who received vaccination against SARS-CoV-2 with a two-dose mRNA vaccine between December 28th 2020 and June 30th 2021, either BNT162b2 (BionTech/Pfizer) or mRNA-1273 (Moderna), given according to the manufacturer’s recommendations. The type of vaccine was assigned according to the available vaccines at the local vaccination point or at our center.

Patients were classified as having had a prior COVID-19 infection if there was clear medical documentation with a positive SARS-CoV-2 PCR swab or in presence of nucleocapsid-IgG specific antibodies in serological analyses before the administration of the first dose of the vaccine. Patients who had received the vaccination schedule before starting HD, patients with an unknown serological status for SARS-CoV-2 before vaccination and patients who either received an adenoviral vector-based vaccine or did not complete the full SARS-CoV-2 mRNA vaccine schedule were excluded from the study.

The study was conducted according to the guidelines of the Declaration of Helsinki and received the approval of the Ethical Committee for Clinical Investigation of the Hospital Universitario Fundación Alcorcón. All patients signed an informed consent form and approved the use of their anonymized clinical information for medical research purposes.

### Vaccination Schedule Data and Sample Collection

**FIGURE 1:**
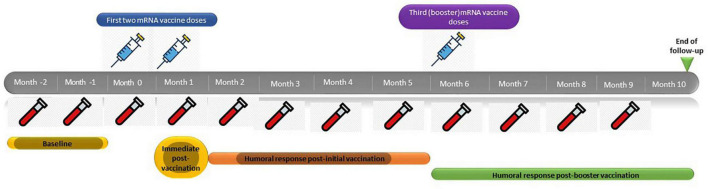
mRNA SARS-CoV-2 vaccine schedule.

Patients received two initial doses of either mRNA vaccine (Pfizer or Moderna) separated by 4 weeks, followed by a third booster dose after 5 months. Serum samples for SARS-CoV-2 anti-spike 1 receptor binding domain [anti-S(RBD)] IgG antibody titers were obtained 2 months prior to the administration of the first dose of the vaccine as well as immediately before vaccination, immediately after the administration of the first and the second dose of vaccine, and thence in a monthly basis up to 4 months after the third booster dose, which would correspond to 10 months after the administration of the first dose (Figure 1). This serological follow-up strategy was performed following the protocol implemented by the Preventive Medicine Department at our center, to be able to assess humoral immunity changes, and detect asymptomatic infected patients.

We examined associations between anti-S(RBD) IgG antibodies and potential predictors such as demographic and clinical data, type of dialysis and type of the vaccine, previous exposure to COVID-19, history of effective humoral response to hepatitis B virus (HBV) vaccines, and treatment with immunosuppressants. Medical histories of study participants were extracted from their medical records.

### Serological Analysis

Antibody response was determined using the chemiluminescent microparticle immunoassay of IgG antibodies to SARS-CoV-2 in human serum (Abbott Laboratories, Chicago, Illinois) which provides quantitative measures of IgG antibodies specific for SARS-CoV-2 S1-RBD. Results are given as arbitrary units per milliliter (AU/ml), and were interpreted as positive according to the manufacturer’s instructions, with a cutoff index value of > 50 AU/ml. The upper limit of quantification was 40,000 AU/ml. The humoral response was considered as effective when the value of SARS-CoV-2 anti-S(RBD) antibodies was > 1,000 AU/ml, which strongly correlates with a sustained neutralizing antibody response of 1/80 measured by plaque reduction neutralization titer (18, 19). The arbitrary units (AU) may be converted to BAU (for the Abbott SARS-CoV-2 IgG II Quant assays the conversion is 1 BAU/mL = 0.142*AU/ml) to harmonize values and find common thresholds for correlates to protection.

### Definitions

Dialysis vintage was defined as the time between the first HD session and the administration of the first dose of mRNA vaccine. Response to HBV vaccine was defined as an anti-HBs antibody titer > 10 IU/ml after four doses of Engerix-B or Fendrix. Immediate humoral response to SARS-CoV-2 vaccines was defined as an anti-S(RBD) antibody titer > 50 AU/ml 4 weeks after the second dose of the vaccine, and the same cutoff was used in every monthly serological assay. Effective humoral response was defined as an anti-S(RBD) antibody titer > 1,000 AU/ml, and sustained humoral response was defined as a persistent antibody titer > 1,000 AU/ml for more than 4 months.

### Statistical Analysis

Demographic and clinical information are described for all patients included in the study. Data were presented as a number (percentage) for categorical variables, as mean ± standard deviation for continuous variables that were normally distributed, and as median (interquartile range; IQR) for continuous variables that were non-parametric. Continuous variables were first tested for normal distribution using Shapiro-Wilk, and then compared by *t*-test, if normally distributed, or by the Mann-Whitney test if abnormally distributed. A Chi-square test was used for categorical variables. Multivariable logistic stepwise regression was used to determine the independent factors associated with an effective humoral response [anti-S(RBD) IgG > 1,000 AU/ml] 4 months after the first two mRNA doses and 4 months after the third dose. The covariates of interest were selected based on the associations with serological response in univariate analyses, and any variables that were at the significance level p less than 0.10 in univariate analyses were included in these models. *P*-value of < 0.05 was considered statistically significant. Statistical analysis was performed with IBM SPSS Statistics for Windows, Version 22.0 (Armonk, NY: IBM Corp.).

## Results

### Patient Characteristics

**FIGURE 2:**
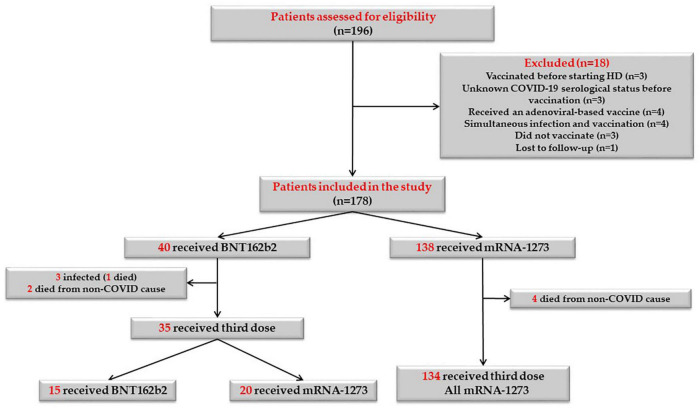
Flowchart of the study population.

One hundred and ninety-six patients on maintenance HD were screened for this study, 18 patients did not meet the inclusion criteria and were therefore excluded. Ultimately, 178 patients were eligible and were included in the study. Figure 2 shows the flowchart of the study population. 138 patients (77.5%) received mRNA-1273 vaccines and 40 patients (22.5%) received BNT162b2 vaccines. 23 patients (12.9%) were considered immunosuppressed; eight patients (34.8%) received anti-cancer treatment and/or targeted therapies, 10 patients (43.5%) were under prolonged steroid treatment, two patients (8.7%) were under treatment with interleukin inhibitors, and three patients (13%) were under treatment with calcineurin inhibitors, one of them with additional mycophenolic acid and steroids. Baseline demographic and clinical characteristics of the whole study population, as well as laboratory and serological data before vaccination are described in Table 1.

**TABLE 1:** Baseline characteristics of the study population and anti-S(RBD) levels follow-up.

	Global cohort (*n* = 178)
Age. years	68.7 ± 14.5
Male gender, *n* (%)	113 (63.5)
Body mass index, kg/m^2^	26.3 ± 5.2
Dialysis vintage, years	3.2 (0.9–7.4)
Type of hemodialysis	
High-flux hemodialysis, *n* (%)	67 (37.6)
On-line hemodiafiltration, *n* (%)	111 (62.4)
Neoplasms, *n* (%)	27 (15.2)
Diabetes mellitus, *n* (%)	73 (41)
Immunosuppressants, *n* (%)	23 (12.9)
HBV vaccine response, *n* (%)	84/122 (68.9)
Previous SARS-CoV-2 exposure, *n* (%)	33 (18.5)
Previous severe COVID-19 that required hospitalization	10 (5.6)
Hemoglobin, g/dl	11.6 ± 1.7
Leukocytes, cells/μl	6,366 ± 2,202
Lymphocytes, cells/μl	1,220 ± 518
Serum albumin, g/dl	3.7 ± 0.4
C- reactive protein, mg/l	4.6 (1.2–13.8)
Baseline anti-S(RBD) IgG levels (AU/ml)	0 (0–2.5)
Positive, *n* (%)	28 (15.7)
> 1,000 AU/ml, *n* (%)	17 (9.6)
Type of vaccine	
Moderna mRNA-1273, *n* (%)	138 (77.5)
Pfizer-BioNTech BNT162b2, *n* (%)	40 (22.5)
Peak anti-S(RBD) IgG levels after two-dose mRNA vaccine (AU/ml)	4,366 (1,271–17,125)
Anti-S(RBD) IgG levels 4 months after the initial two-dose mRNA vaccine (AU/ml)	1,144 (232–3,598)
Type of booster vaccine	
Moderna mRNA-1273, *n* (%)	155/169 (91.7)
Pfizer-BioNTech BNT162b2, *n* (%)	14/169 (8.3)
Peak anti-S(RBD) IgG levels after booster dose (AU/ml)	26,037 (11,976–40,000)
Anti-S(RBD) IgG levels 4 months after booster dose (AU/ml)	9,180 (3,489–30,499)

### Humoral Response After Vaccination in the Whole Study Population

**FIGURE 3:**
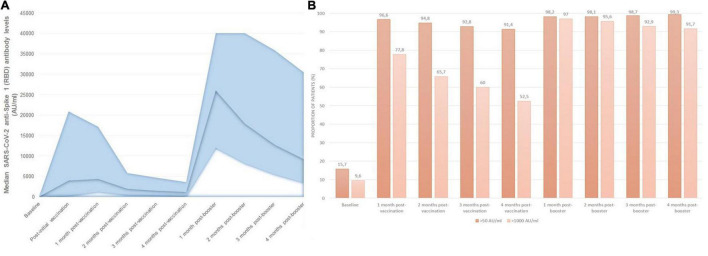
Monthly SARS-CoV-2 serological follow-up: **(A)** median monthly anti-S(RBD) antibody levels, **(B)** proportion of patients with positive and effective humoral responses at each time point during follow-up.

The evolution of IgG anti-S(RBD) antibody levels in the whole cohort throughout the study period is represented in Figure 3. After receiving the second dose of mRNA vaccine, 96.6% of patients had positive anti-S(RBD) antibodies, with a gradual decrease in the proportion of patients with positive antibodies to 91.4% after 4 months from the second dose. Median anti-S(RBD) antibody levels decreased from a peak of 4,366 AU/ml (1,271–17,125) 1 month after the second vaccine dose, to 1,144 AU/ml (232–3,598) after 4 months (*p* < 0.001). After receiving the third mRNA vaccine dose, the proportion of patients who seroconverted increased to 98.3%, and this rate remained stable for the following 4 months. Median antibody levels rose up to 26,037 AU/ml (11,976–40,000) and decreased to 9,180 AU/ml (3,489–30,499) after 4 months from the booster dose (*p* < 0.001).

The proportion of patients with IgG anti-S(RBD) antibody levels over 1,000 AU/ml changed in a similar manner, from 77.8% of patients after receiving the first two doses, to 52.5% of patients 4 months after the second dose, rising up to 97% after receiving the third booster dose, and remaining in 91.7% of patients after 4 months from the third dose (Figure 3B). Overall, patients receiving mRNA-1273 had higher peak anti-S(RBD) antibody levels compared with BNT162b2 [median 6,468 AU/ml (IQR 1927-19623) vs. 1,630 AU/ml (IQR 482-3554), *p* < 0.001].

### Changes in Humoral Response According to Previous Exposure to SARS-CoV-2

We stratified the study population according to previous SARS-CoV-2 exposure into patients with prior COVID-19 infection before vaccination (*n* = 33) and those who were COVID-19 naïve at the time of vaccination (*n* = 145) (Table 2). There was a higher proportion of males (78.8% vs. 60%, *p* = 0.04), and lower proportion of patients with online haemodiafiltration (45.5% vs. 66.2%, *p* = 0.03) in the group of patients with prior COVID-19 infection. Pre-vaccination levels of anti-S(RBD) IgG antibodies were elevated in 78.8% of COVID-exposed patients, with a median antibody titer of 1,123 AU/ml, of which 51.5% had antibody levels higher than 1,000 AU/ml before vaccination. On the other hand, in the COVID-naïve group only two patients (1.4%) had positive anti-S(RBD) antibody levels that were slightly elevated (56 and 63 AU/ml) with negative IgG nucleocapsid antibodies and no history of COVID-19 infection, and were thus considered COVID-naïve.

**TABLE 2:** Baseline characteristics and anti-S(RBD) levels follow-up stratified according to previous SARS-CoV-2 exposure: and HBV vaccine response status.

	Prior COVID-19 infection (*n* = 33)	COVID-naïve (*n* = 145)	*P*	HBV vaccine responders (*n* = 84)	HBV vaccine non-responders (*n* = 38)	*P*
Age. years	65.7 ± 16.2	69.1 ± 14.1	0.188	67.5 ± 14.1	70.2 ± 15	0.341
Male gender, *n* (%)	26 (78.8)	87 (60)	0.043	52 (61.9)	22 (57.9)	0.675
Dialysis vintage. years	2.4 (0.6–7.1)	3.5 (1.2–7.5)	0.155	3.7 (0.9–7.8)	4.3 (2.4–9.8)	0.111
Type of hemodialysis						
High-Flux hemodialysis, *n* (%) On-line hemodiafiltration, *n* (%)	18 (54.5) 15 (45.5)	49 (33.8) 96 (66.2)	0.026	24 (28.6) 60 (71.4)	18 (47.4) 20 (52.6)	0.043
Neoplasms, *n* (%)	3 (9.1)	24 (16.6)	0.214	6 (7.1)	9 (23.7)	0.010
Diabetes mellitus, *n* (%)	17 (51.5)	56 (38.6)	0.174	34 (40.5)	17 (44.7)	0.659
Immunosuppressants, *n* (%)	5 (15.2)	18 (12.4)	0.672	7 (8.3)	9 (23.7)	0.020
HBV vaccine response, *n* (%)	15/19 (78.9)	69/103 (67)	0.301			
Prior COVID-19 exposure, *n* (%)				15 (17.9)	4 (10.5)	0.301
Baseline anti-S(RBD) IgG levels (AU/ml)	1,123 (124–2,451)	0 (0–0.1)	<0.001	0 (0–1.9)	0 (0–0.2)	0.203
Positive, *n* (%)	26 (78.8)	2 (1.4)	2 (1.4)	10 (12.2)	4 (10.8)	0.548
> 1,000 AU/ml, *n* (%)	17 (51.5)	0	<0.001	6 (7.8)	3 (8.1)	0.572
Type of vaccine Moderna mRNA-1273, *n* (%) Pfizer-BioNTech BNT162b2, *n* (%)	26 (78.8) 7 (21.2)	113 (77.9) 32 (22.1)	0.914	69 (82.1) 15 (17.9)	29 (76.3) 9 (23.7)	0.453
Peak anti-S(RBD) IgG levels after two-dose mRNA vaccine (AU/ml)	40,000 (23,553–40,000)	3,061 (950–8,999)	<0.001	5,003 (2,276–16,576)	2,056 (444–8,223)	0.002
Anti-S(RBD) IgG levels 4 months after the initial two-dose mRNA vaccine (AU/ml)	26,390 (5,773–40,000)	670 (192–2,030)	<0.001	1,361 (481–3,598)	341 (120–1,548)	0.001
Type of booster vaccine						
Moderna mRNA-1273, *n* (%) Pfizer-BioNTech BNT162b2, *n* (%)	27/30 (90) 3/30 (10)	128/139 (92.1) 11/139 (7.9)	0.468	73 (91.2) 7 (8.8)	35 (94.6) 2 (5.4)	0.414
Peak anti-S(RBD) IgG levels after booster dose (AU/ml)	40,000 (21,872–40,000)	22,419 (9,356–40,000)	<0.001	28,845 (17,876–40,000)	16,587 (7,449–40,000)	0.048
Anti-S(RBD) IgG levels 4 months after booster dose (AU/ml)	29,740 (11,637–40,000)	6,970 (2,907–22,903)	<0.001	9,760 (4,565–30,421)	4,731 (1,666–17,593)	0.010

**FIGURE 4:**
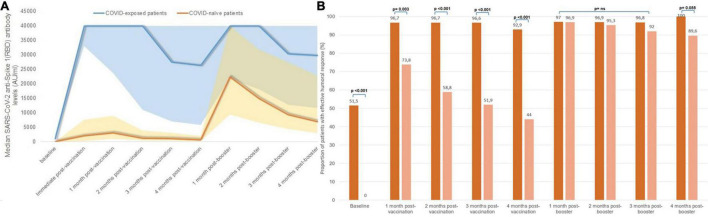
Serological changes according to SARS-CoV-2 exposure status: **(A)** median monthly anti-S(RBD) antibody levels, **(B)** proportion of patients with effective humoral response after vaccination according to prior SARS-CoV-2 exposure.

The proportion of patients who achieved seroconversion was similar in both groups (Supplementary Figure 1). However, the evolution of antibody levels was significantly different in both groups; peak IgG anti-S(RBD) antibody levels after the first two vaccine doses were higher in patients with a prior exposure to COVID-19 (median 40,000 AU/ml, IQR 23,553–40,000) compared with those who were COVID-naïve (median 3,061 AU/ml, IQR 950–8,999) (*p* < 0.001), and remained so at every monthly assay. Serological changes in patients with previous COVID-19 exposure and in COVID-naïve patients is represented in Figure 4A. Although there were significant differences in the proportion of patients with an effective seroconversion of anti-S(RBD) levels > 1,000 AU/ml between COVID-naïve and COVID-exposed patients following the initial two-dose mRNA vaccination, the administration of a third mRNA vaccine dose matched the proportion of effective humoral response between both groups (Figure 4B). However, median antibody levels remained significantly higher in patients previously exposed to COVID-19 compared to COVID-naïve patients throughout the study period, even after receiving the third vaccine dose (at end of follow-up: 29,740 AU/ml [11,637–40,000] vs. 6,970 AU/ml [2,907–22,903], *p* < 0.001).

### Changes in Humoral Response According to Previous Response to Hepatitis B Virus Vaccines

**FIGURE 5:**
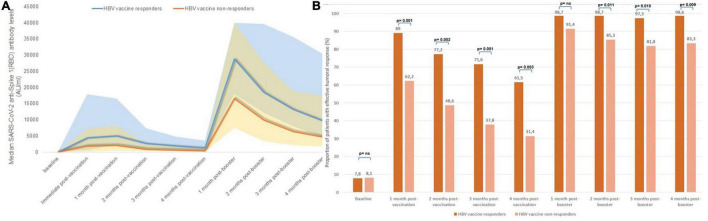
Serological changes according to previous response to HBV vaccines: **(A)** median monthly anti-S(RBD) antibody levels, **(B)** Proportion of patients with effective humoral response after vaccination according to response to HBV vaccine.

In our cohort, 122 patients (68.5%) had received a full HBV vaccination regimen, of which 84 patients (68.9%) had an effective humoral response. Table 2 shows the baseline differences between HBV vaccine responders and non-responders, and differences in median antibody levels throughout follow-up. HBV vaccine responders showed a significantly higher initial humoral response to SARS-CoV-2 mRNA vaccines compared to those patients who previously did not respond to HBV vaccines (Figure 5A). Peak IgG anti-S(RBD) antibody levels were higher in patients with a history of effective humoral response to HBV vaccines (median 5,003 AU/ml, IQR 2,276–16,576) compared with HBV vaccine non-responders (median 2,056 AU/ml, IQR 444–8,223) (*p* = 0.002), and these differences remained for the following 4 months after the initial two-dose vaccine. Nonetheless, after the third SARS-CoV-2 vaccine dose, the proportion of patients who had positive anti-S(RBD) antibody levels was matched in both groups (Supplementary Figure 2), but the rate of effective humoral response after the third booster dose remained significantly higher in HBV vaccine responders from the second month onward (Figure 5B).

### Decrease in Anti-S(RBD) Antibody Levels Before and After the Third mRNA Vaccine Dose

**FIGURE 6:**
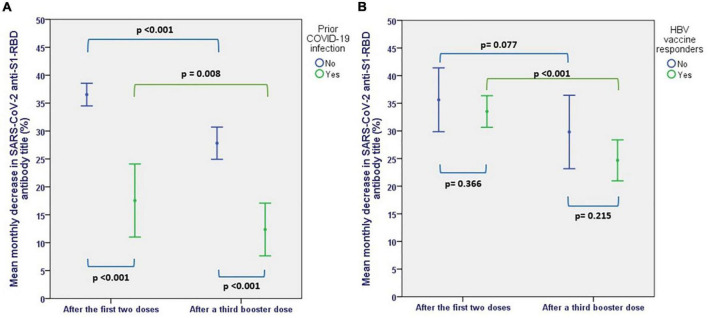
Mean monthly decrease in SARS-CoV-2 AntiS(RBD) antibodies before and after a third booster dose, **(A)** according to prior SARS-CoV-2 exposure, **(B)** according to response to HBV vaccines.

After a median follow-up of 4 months after the second dose of mRNA vaccine, there was a mean monthly decrease in anti-S(RBD) antibodies of 33 ± 14.5%. This decrease was significantly steeper in patients who were COVID-naïve (36.5 ± 11% per month) compared to patients with previous exposure to COVID-19 (17.5 ± 6%) (*p* < 0.001) (Figure 6A).

The mean monthly rate of antibody titer decline decreased to 25 ± 16.7% after the third dose (*p* < 0.001). Although the rate of antibody level decline remained significantly lower in patients with previous exposure to COVID-19 (12.4 ± 8% vs. 27.8 ± 16%, *p* < 0.001), COVID-naïve patients also showed a more sustained humoral response after the third dose compared to the initial vaccination, with a monthly decrease in the rate of antibody level decline from 36.5 ± 11 to 27.8 ± 16% (*p* < 0.001). Also, patients who were responders to HBV vaccines showed a more sustained response after the third dose, with a decrease in the rate of antibody decline from 33.5 ± 12.3 per month to 24.7 ± 16% per month (*p* < 0.001) (Figure 6B).

### Determinants of Sustained Seroconversion After the Initial Vaccination

We analyzed factors that were associated with an effective humoral response after the initial two-dose mRNA vaccination. Patients with previous exposure to COVID-19 (OR 16.5, CI 95% 3.8–72.5), previous humoral response to HBV vaccines (OR 3.5, CI 95% 1.5–8.1), patients who were < 70 years old (OR 3.5, CI 95% 1.8–6.7) and patients who received mRNA-1273 (OR 3.2, CI 95% 1.4–7.3) were associated with an effective sustained humoral response after 4 months from vaccination in the univariate analysis. Patients with history of neoplasms were less likely to maintain an effective humoral response (OR 0.4, CI 95% 0.2–1.1). In the multivariable linear regression model, both previous exposure to COVID-19 infection and previous humoral response to HBV vaccines maintained statistical significance. Details are presented in Table 3.

**TABLE 3:** Univariate and multivariate logistic regression for predicting sustained effective humoral response (SARS-CoV-2 anti-S-RBD antibody levels > 1,000 AU/ml) 4 months after the initial two mRNA doses.

	Univariate analysis				Multivariate analysis			
	OR	95% confidence interval Lower limit upper limit		*P*	OR	95% Confidence interval Lower limit upper limit		*P*
Age < 70 years	3.48	1.82	6.67	<0.001	2.12	0.86	5.21	0.103
Male gender	1.00	0.53	1.89	0.998				
Dialysis vintage < 3 years	1.51	0.81	2.81	0.194				
On-line hemodiafiltration (vs. high-flux HD)	1.61	0.85	3.08	0.147				
Neoplasms	0.43	0.17	1.08	0.072	0.91	0.25	3.35	0.884
Diabetes	0.94	0.50	1.77	0.852				
Immunosuppressant treatment	0.64	0.25	1.62	0.347				
HBV vaccine response (Ac HBs > 10)	3.49	1.50	8.14	0.004	3.36	1.31	8.62	0.012
Prior COVID-19 infection	16.53	3.77	72.46	<0.001	8.85	1.59	49.47	0.013
Type of vaccine Moderna mRNA1273 (vs. Pfizer-BioNTech BNT162b2)	3.19	1.41	7.26	0.006	3.46	0.93	12.93	0.065

### Determinants of Sustained Seroconversion After the Booster Dose

In the univariate analysis to determine the factors associated with a sustained humoral response after 4 months from receiving the third dose of mRNA vaccine, we found an association with response to HBV vaccines (OR 13.8, CI 95% 1.5–123.9), and a tendency for a less likely effective seroconversion in patients receiving immunosuppressants (OR 0.29, CI 95% 0.08–1.08). After adjusting for age, dialysis vintage, immunosuppression, and type of vaccine administered, the humoral response to HBV vaccines remained as an independent factor associated with an effective sustained humoral response after 1 year from the initial vaccination. All patients with prior exposure to COVID-19 developed an effective humoral response, and therefore could not be included as a factor in the multivariate analysis (Table 4).

**TABLE 4:** Univariate and multivariate logistic regression for predicting sustained effective humoral response (SARS-CoV-2 anti-S-RBD antibody levels > 1,000 AU/ml) 4 months after the third mRNA booster dose.

	Univariate analysis				Multivariate analysis			
	OR	95% confidence interval Lower limit upper limit		*P*	OR	95% Confidence interval Lower limit upper limit		*P*
Age < 70 years	2.87	0.74	11.06	0.126	0.99	0.14	6.91	0.994
Male gender	0.79	0.24	2.63	0.701				
Dialysis vintage < 3 years	3.44	0.89	13.26	0.073	4.15	0.40	42.74	0.231
On-line hemodiafiltration (vs. high-flux HD)	0.83	0.24	2.90	0.770				
Neoplasms	0.47	0.12	1.90	0.289				
Diabetes	0.90	0.27	2.98	0.862				
Immunosuppressant treatment	0.29	0.08	1.08	0.065	0.21	0.03	1.40	0.108
HBV vaccine response (Ac HBs > 10)	13.8	1.54	123.9	0.019	9.63	1.01	92.56	0.049
Prior COVID-19 infection	Constant 8.58			<0.001				
Type of vaccine Moderna mRNA1273 (vs. Pfizer-BioNTech BNT162b2)	2.80	0.83	9.53	0.098	0.87	0.06	11.71	0.913

### Breakthrough COVID-19 Infections

Throughout the year following SARS-CoV-2 vaccination, there were 23 vaccinated patients (12.9%) who were diagnosed with SARS-CoV-2 infection, of which 3 cases (1.7%) were after receiving the first two doses, and the remaining cases (11.2%) were diagnosed after receiving the third dose of vaccination. Twenty-one patients (91.3% of the infected patients) remained asymptomatic. There were only two cases (1.1%) of severe COVID-19 with acute respiratory failure requiring hospital admission and high flow oxygen therapy among vaccinated patients included in this study, both of which had an inadequate humoral response to the vaccine. The first patient was an 85-year-old male patient who became infected 3 months after receiving the first two mRNA vaccine doses, having reached a peak SARS-CoV-2 anti-S(RBD) antibody titer of 1,007 AU/ml in the first month after vaccination, and decreasing to 510 AU/ml in the second month, just before infection. The second patient was a 72-year-old female patient under immunosuppression with tocilizumab due to severe rheumatoid arthritis. She never responded to the initial two doses of mRNA vaccine nor to the booster dose (peak SARS-CoV-2 anti-S-RBD antibodies 1 AU/ml) and was infected 3 months after receiving the third dose. Neither patient had a history of COVID-19 infection before vaccination, and both patients died.

## Discussion

In this serological study we examined the monthly changes in SARS-CoV-2 anti-S(RBD) IgG levels for 1 year after vaccination. We observed that patients who were COVID-naïve and those who had not previously responded to HBV vaccines benefited the most from a third mRNA vaccine dose against SARS-CoV-2, since it provided them with an effective humoral response (anti-S-RBD IgG levels > 1,000 AU/ml), both immediately after vaccination and also sustained in time for at least 4 months after receiving the booster dose, whereas the first two doses had been unable to elicit such a sustained response.

Several studies have determined the factors that are associated with an immediate humoral response few weeks after vaccination, including serum albumin levels, lymphocytes, dialysis vintage, intravenous iron dose, immunosuppressant treatment, previous infection with COVID-19, and a positive response to HBV vaccines (20–22). In our study, we demonstrate in a multivariable model that the factors associated with a sustained humoral response 4 months after the third booster dose were having had a previous infection with COVID-19 and an effective response to HBV vaccines.

Naïve patients for COVID-19 infection are the ones who benefit the most from vaccination and especially from the administration of a third dose (11). In our study, the serological changes in COVID-naïve patients following vaccination is similar to that previously described: a high proportion of patients (96.8%) develop SARS-CoV-2 anti-S(RBD) antibodies after the first two doses of the vaccine, but not all patients develop an effective response. Only 77.8% of patients achieved a protective response of > 1,000 AU/ml which decreased to 52.5% after 4 months, with a mean decrease of 33% per month in antibody levels. It has been previously described that, thanks to the booster effect, there is an improvement in the humoral response in the first weeks, especially in COVID-naïve patients who did not have an initial response (12, 16, 17, 23). In our study this improvement persists for 4 months after the booster effect. After the third dose, the proportion of patients with antibody levels > 1,000 AU/ml immediately increased to 97%, and the mean monthly drop in antibody levels significantly decreased compared to that following the first two doses, from 36.5 to 27.8% (*p* = 0.009). In this way, 4 months after receiving the booster dose, there were no significant differences in the protective humoral response between COVID-naïve patients and those who were previously infected with COVID-19 (89.7% vs. 100%, *p* = 0.053).

During the study period, the accumulated incidence in our area was around 20,500 cases per 100,000 population (24), predominantly caused by the omicron variant (25). We can assume that these high levels of antibodies provided our patients with an effective protection, as is witnessed by the excellent response in those patients who became infected during follow-up, most of whom were asymptomatic or had mild symptoms. In this respect, the beneficial effects of a third dose of mRNA vaccines to achieve this effective serological response against the new variants have been described in the general population (26) and it is probably associated with attaining high levels of antibodies (27).

HD patients who have had a COVID-19 infection have less protection against reinfection than the general population (28). However, 10–14 days after administering the second dose of mRNA vaccine, the serological response was similar to that of healthy vaccinated patients. For this reason, it has been suggested that they would not benefit from the administration of a third dose (12). In our population of HD patients who were previously infected with COVID-19, the initial two-dose mRNA vaccine achieved an increase in effective humoral response from 54.8 to 96.8%, decreasing to just 93.1% after 4 months. Following the third mRNA vaccine dose, all the previously infected patients achieved SARS-CoV-2 anti-S(RBD) antibody levels > 1,000 AU/ml after 4 months from vaccination, and the mean decrease in antibody levels decreased from 17.5% following the second dose to 12.4% after the third dose (*p* < 0.001). Therefore, this group of patients not only benefits from the first two vaccine doses but also from the booster effect of a third dose, although to a lesser extent than in the COVID-naïve population.

Previous studies have compared the humoral response after the second and third doses. One study analyzed the humoral response after the third dose of the vaccine, showing a slight increase in the proportion of patients who achieve seroconversion, but with a very intense humoral response, reaching a 36-fold increase in median antibody titers after the booster dose (29), and another study showed that a third dose reduced the proportion of patients with no or weak response from 60.8 to 15% (30). An additional study determined that 6 months after the first immunization, 96% of patients who had received three vaccine doses had responded positively, compared to 64% when only two doses had been administered (31). The rate of immediate seroconversion observed in our study after the booster dose confirms the data described by these previous studies. In addition, we verified that the number of seroconverted patients remains constant (99%) after 4 months, most of whom show a protective humoral response (91.7%).

It has been previously described that there is an association between humoral responses after HBV and SARS-CoV-2 vaccines in HD (21, 32, 33). In our study, humoral response to HBV vaccines was an independent factor associated with a sustained humoral response after SARS-CoV-2 vaccination. Similar to COVID-naïve patients, those patients who had not previously responded to HBV vaccines have a worse response to immunization with two doses of mRNA vaccine and clearly benefit from the booster effect. Accordingly, HD patients who are non-responders to HBV vaccines warrant a close serological follow-up against SARS-CoV-2 to monitor immune response and would possibly benefit from a more intense vaccination schedule. Although in previous studies immunosuppressive therapy was shown to be a factor predicting poor response (20), in our study we did not find an association between immunosuppression and long-term humoral response following a third vaccine dose after adjusting for confounding variables.

It should be noted that the most used vaccine in our patients was mRNA-1273, 77.5% of the patients for the first two doses and 91.7% for the third dose (with no significant differences between the different groups of patients). It has been described that the type of vaccine acts as an independent factor for response to vaccination and that mRNA-1273 is the one that obtains the best results, perhaps due to its higher dose of mRNA (21).

We believe that in the future, the rate of antibodies will continue to decline and a significant percentage of patients will probably lose protection in the mid-term. Therefore, monitoring the levels of antibodies in the HD population should be a priority in order to propose new immunizations when necessary, as other authors have suggested (34).

Consequently, we could revaccinate when SARS-CoV-2 anti-S(RBS) IgG levels drop below a certain threshold, in a similar way to how revaccination of HBV is recommended when anti-HBs antibodies drop below 10 mIU/mL. We suggest that monitoring of anti-S(RBD) IgG levels should be particularly frequent in patients who did not respond to HBV vaccines, in COVID-naïve patients and in those with lower antibody levels.

Our study has a number of limitations. First, the rate of neutralizing antibodies was not determined, which according to other authors can decrease progressively after the first two doses and can rise very significantly after the third dose. However, other studies have found a significant correlation between anti-S(RBD) antibodies and the neutralizing antibody response, and in our study we found that the changes in anti-S(RBD) IgG antibody levels resemble those described in neutralizing antibodies (18, 19). Another limitation to this study is that cellular immunity was not assessed. It has been described that the response of cellular immunity to vaccination also improves after the third dose (20), and some authors have suggested as a potential predictor of the response to a third dose of the vaccine (12). Since circulating antibody titers are not predictive of T cell memory (35), serological tests for SARS-CoV-2 antibodies may not reflect the strength and longevity of the immune memory to SARS-CoV-2. Finally, the limited number of cases prevents us from drawing solid conclusions about the efficacy of protection against reinfection and its severity (hospitalization and mortality).

## Conclusion

In conclusion, as a consequence of receiving a third dose of SARS-CoV-2 mRNA vaccine, HD patients achieve a better and more sustained humoral immune response over time, which probably translates into lower morbidity and mortality rates in SARS CoV-2 infection. Patients who are most likely to benefit from the booster dose are COVID-naïve patients and patients who were non-responders to HBV vaccines.

## Data Availability Statement

The raw data supporting the conclusions of this article will be made available by the authors, without undue reservation.

## Ethics Statement

The studies involving human participants were reviewed and approved by the Medical Research Ethical Committee (CEIm), Hospital Universitario Fundación Alcorcón. The patients/participants provided their written informed consent to participate in this study.

## Author Contributions

EG-V and AS: conceptualization, writing—original draft preparation, and writing—review and editing. EG-V: methodology and project administration. EG-V, AS, and ML-P: validation. AS: formal analysis. JA-S: investigation. EG-V and EG: resources. EG-V and ML-P: data curation. JA-S, AC, CC-C, and GF-J: visualization. All authors have read and agreed to the published version of the manuscript.

## Conflict of Interest

The authors declare that the research was conducted in the absence of any commercial or financial relationships that could be construed as a potential conflict of interest.

## Publisher’s Note

All claims expressed in this article are solely those of the authors and do not necessarily represent those of their affiliated organizations, or those of the publisher, the editors and the reviewers. Any product that may be evaluated in this article, or claim that may be made by its manufacturer, is not guaranteed or endorsed by the publisher.

## Supplementary Material

Supplementary Figure 1 Proportion of patients with positive SARS-CoV-2 anti-S(RBD) IgG antibodies throughout the study period, according to prior SARS-CoV-2 exposure.

Supplementary Figure 2 Proportion of patients with positive SARS-CoV-2 anti-S(RBD) IgG antibodies throughout the study period, according to response to HBV vaccines.

Supplementary Table 1 Mean monthly % decrease in anti-S(RBD) levels.
