# Supplementary figures and images for: Humoral Response Following Triple Dose of mRNA Vaccines Against SARS-CoV-2 in Hemodialysis Patients: Results After 1 Year of Follow-Up
Source: Front Med (Lausanne). 2022 Jul 12;9:927546. doi: 10.3389/fmed.2022.927546 (PMC9314744; doi:10.3389/fmed.2022.927546)

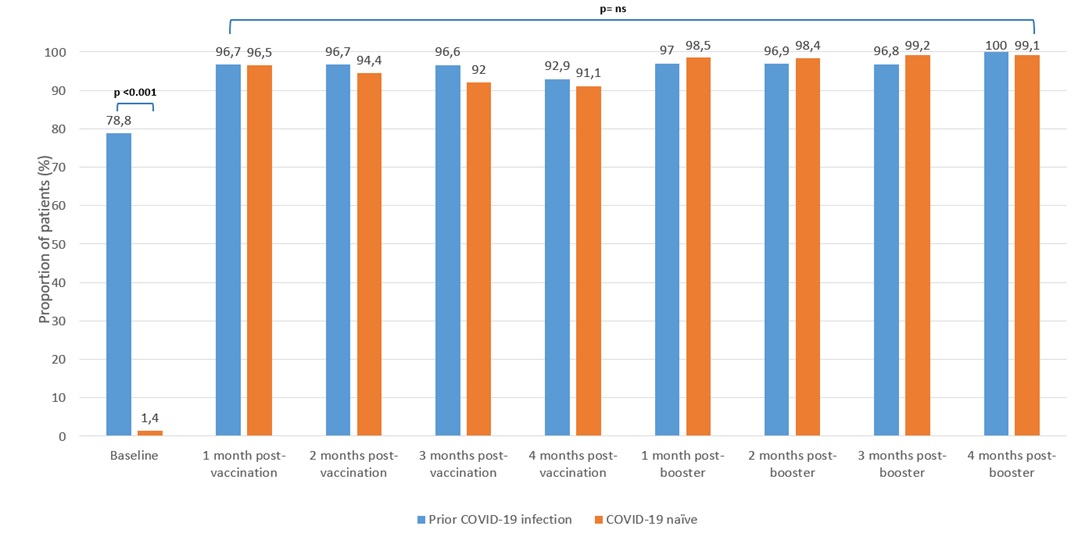

Supplement: Supplementary Figure 1 — Proportion of patients with positive SARS-CoV-2 anti-S(RBD) IgG antibodies throughout the study period, according to prior SARS-CoV-2 exposure. [file Image_1.JPEG]

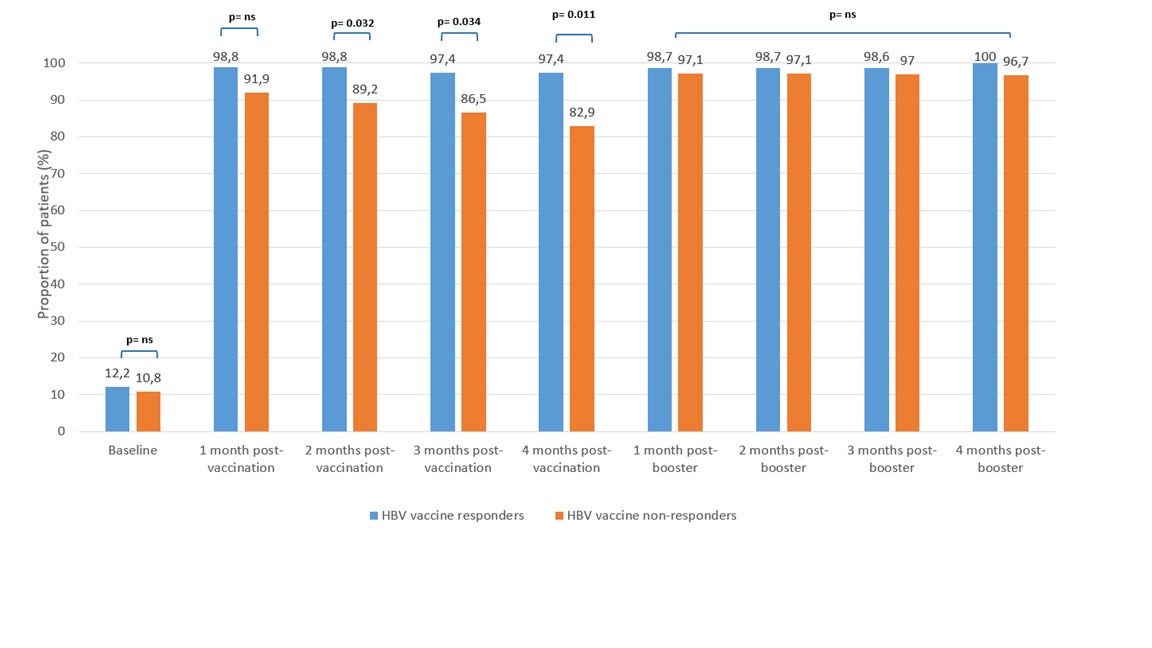

Supplement: Supplementary Figure 2 — Proportion of patients with positive SARS-CoV-2 anti-S(RBD) IgG antibodies throughout the study period, according to response to HBV vaccines. [file Image_2.JPEG]
