# Supplementary material for: Humoral Response Following Triple Dose of mRNA Vaccines Against SARS-CoV-2 in Hemodialysis Patients: Results After 1 Year of Follow-Up
Source: Front Med (Lausanne). 2022 Jul 12;9:927546. doi: 10.3389/fmed.2022.927546 (PMC9314744; doi:10.3389/fmed.2022.927546)
Supplement: Supplementary Table 1 — Mean monthly % decrease in anti-S(RBD) levels. [file Table_1.docx]

Supplementary table 1. Mean monthly % decrease in anti-S(RBD) levels

|  | Before the third booster dose | After the third booster dose | p |
| --- | --- | --- | --- |
| Global cohort | **33 ± 14.5** | **24.8 ± 16.7** | **<0.001** |
| prior COVID-19 infection | **17.5 ± 6.2** | **12.4 ± 8.2** | **<0.001** |
| COVID-19 Naïve | **36.5 ± 11.4** | **27.8 ± 16.3** | **0.009** |
| HBV vaccine responders | **33.5 ± 12.3** | **24.7 ± 16** | **<0.001** |
| HBV vaccine non-responders | **35.6 ± 16** | **29.8 ± 18.4** | **0.077** |
